# Hantavirus Seroprevalence in the Population of Saint Petersburg and the Leningrad Region, Russia

**DOI:** 10.3390/v18060652

**Published:** 2026-06-06

**Authors:** Tatiana Arbuzova, Dmitry Naydenov, Regina Baimova, Alena Khalilova, Denis Sarksyan, Konstantin Manakhov, Tamara Ginevskaia, Margarita Popova, Ekaterina Klyuchnikova, Svetlana Egorova, Vladimir Dedkov, Areg Totolian

**Affiliations:** 1 Saint Petersburg Pasteur Institute, 197101 Saint Petersburg, Russiatotolian@spbraaci.ru (A.T.); 2 Izhevsk State Medical University, 426034 Izhevsk, Russia; 3 Martsinovsky Institute of Medical Parasitology, Tropical and Vector Borne Diseases, Sechenov First Moscow State Medical University, 119048 Moscow, Russia

**Keywords:** seroprevalence, hantavirus, hemorrhagic fever with renal syndrome

## Abstract

The aim of the study was to assess the seroprevalence of hantaviruses, the causative agents of hemorrhagic fever with renal syndrome (HFRS), and their distribution to socio-demographic characteristics among the populations of Saint Petersburg and the Leningrad Region. A total of 4464 samples were analyzed, including 2265 samples from residents of Saint Petersburg and 2199 samples from residents of the Leningrad Region. Blood plasma samples were tested for specific immunoglobulin G (IgG) antibodies using enzyme-linked immunosorbent assay (ELISA). Blood samples were collected in 2023 from randomly selected volunteers. Hantavirus seroprevalence in Saint Petersburg was 5.39%, while in the Leningrad Region, it was 8.55%. In both regions, the highest proportion of seropositive individuals was found among volunteers aged ≥70 years, whereas the lowest seroprevalence was observed in the 1–17-year age group (inclusive). Seroprevalence was significantly higher in men than in women in both regions. The seroprevalence values identified in this study are comparable to those reported in similar studies in areas with a high incidence of HFRS. These findings may indicate that the true incidence of HFRS may be significantly higher than officially registered in Saint Petersburg and the Leningrad Region.

## 1. Introduction

In Russia, hemorrhagic fever with renal syndrome (HFRS) is the most prevalent natural focal viral disease; vaccine development efforts are currently underway, although no officially approved vaccination against this disease is currently available [1,2]. HFRS pathogens belong to the order *Bunyavirales*, family *Hantaviridae*, which includes the genus *Orthohantavirus*. Hantaviruses exhibit high genetic diversity and their geographic distribution closely correlates with the ecological ranges of natural reservoirs (rodents) [2,3]. The primary transmission route for hantaviruses is airborne dust. Infection occurs via inhalation of aerosols containing excreta, saliva, or urine from infected rodents. Infection risk is particularly elevated in rural and suburban areas, where rodent contact is most likely [4,5,6]. The boundaries of HFRS natural foci are gradually expanding, encompassing regions previously considered free of hantavirus infection [7,8,9]. This may stem from climate change, urbanization, and increased anthropogenic pressure on murine rodent habitats [10].

Russia’s Northwestern Federal District (NWFD) ranks third in HFRS incidence after the Volga and Central Regions [11]. According to official statistics in the report “Information on Infectious Morbidity and Parasitic Diseases,” the mean annual incidence (HFRS) over the study period (2014–2024) was 1.17 per 100K population in the NWFD. In Saint Petersburg (SPb), 482 HFRS cases were reported in 2014–2024, and the mean annual incidence over the study period was 0.83 per 100K population. Regarding the Leningrad Region (LR), 74 HFRS cases were officially reported during 2014–2024; the mean annual incidence over the study period was 0.34 per 100K population. The LR directly borders Finland, an adjacent country with one of the highest reported HFRS incidence levels in Europe [12,13]. In Finland, the reported HFRS incidence ranges from 31 to 39 cases per 100K population annually [14]. Geographic characteristics of the LR may affect hantavirus circulation due to transboundary migration of rodent reservoir hosts, primarily *Myodes glareolus* (bank vole), which is widely distributed in Finland [14].

Bank voles serve as the primary reservoir of Puumala virus (PUUV) and are capable of migrating over considerable distances, thereby facilitating the spread of the pathogen to more remote areas [15]. Officially reported HFRS cases in the NWFD are associated with PUUV, with the exception of sporadic cases caused by Dobrava–Kurkino virus in the LR [16]. Similar climatic and environmental conditions in the NWFD and the Scandinavian countries, including the presence of temperate and boreal forest ecosystems, create favorable conditions to support stable rodent populations and the persistence of natural hantavirus foci. Evidence of hantavirus circulation in regions with a low reported incidence of hemorrhagic fever with renal syndrome (HFRS) is provided by previous serological studies reporting immunoglobulin G (IgG) antibodies in the population [17,18,19,20].

The existence of natural foci in regions characterized by intense socio-economic factors (urbanization, agricultural activity, globalization) highlights the need for coordinated cross-border programs focused on epidemiological surveillance, environmental research, and public health interventions. Such measures would contribute to reducing the risks associated with the expansion of hantavirus circulation within a shared ecological region or across transboundary ecosystems. Assessment of population immunity represents a key component of epidemiological surveillance. It enables assessment of both the risk and the magnitude of the epidemic threat posed by disease spread in Russian regions with endemic HFRS. The aim of this study was to determine hantavirus seroprevalence and its distribution according to socio-demographic characteristics among the population of the SPb and LR.

## 2. Materials and Methods

### 2.1. Study Design and Population

Blood samples were collected in September 2023 from randomly selected volunteers as part of the Rospotrebnadzor program “Assessment of collective immunity to vaccine-preventable and other relevant infections among residents of SPb and the LR” [21]. Participants were randomly selected using a web-based questionnaire, and randomization was performed according to clinical data age group and district. The questionnaire contained personal information, information about chronic diseases, blood transfusions and surgical interventions, vaccination status regarding vaccine-preventable and other relevant infections (with dates). Inclusion criteria were as follows: age > 1 year, individuals who had resided in SPb or the LR for more than one year and who were not undergoing treatment at the time of enrollment; no more than 30 people with the same affiliation (same industrial, educational, or healthcare institution, etc.). The exclusion criteria were the presence of any active infectious diseases (of any etiology) at the time of blood collection and persons with contraindications to blood sampling. All participants were informed about the aims of the study and provided written informed consent to participate in the study. The study protocol was approved by the Local Ethics Committee of the Saint Petersburg Pasteur Institute (protocol 86, 17 August 2023).

A total of 4464 samples were analyzed: 2265 samples from residents of SPb and 2199 from residents of the LR. Sample size was insufficient only in the Kronshtadtsky district (with 10 volunteers examined). In the remaining city districts, sample sizes ranged from 31 to 497 specimens. Among volunteers from the LR, residents of all districts were included (except the town of Sosnovy Bor); the sample size in individual districts ranged from 55 to 141 participants.

Participants were divided into seven age groups: 1–17 years, 18–29 years, 30–39 years, 40–49 years, 50–59 years, 60–69 years, and ≥70 years. The size of the age groups ranged from 270 to 362 individuals (Table 1). Among all participants, 1335 (29.91%) were male, and 3129 (70.09%) were female.

### 2.2. Serological Testing

To assess hantavirus seroprevalence in SPb and the LR, blood plasma was analyzed for the presence of hantavirus-specific IgG antibodies using a solid-phase enzyme immunoassay (ELISA) with the commercial VectoHantaIgG reagent kit (Vector-Best, Novosibirsk, Russia), which holds a registration certificate and is intended for the clinical laboratory diagnosis of hantavirus infection in human serum or plasma. Results were considered positive if the optical density in the sample exceeded the cut-off value calculated according to the formula provided in the manufacturer’s instructions. Blood sampling was performed from the cubital vein, with 3 mL collected into vacutainers containing K_3_EDTA. The vacutainers were centrifuged for 10 min at 1000 rpm at room temperature. Blood plasma was separated from cellular components, transferred into microcentrifuge tubes, and stored at 4 °C until testing.

### 2.3. Statistical Analysis

For the analysis of categorical variables and identification of statistically significant differences in seroprevalence between sex and age groups, the chi-square (χ^2^) test was applied [22]. Differences were considered statistically significant at *p* < 0.05 [23]. Correlation analysis was performed depending on data distributions using Spearman’s rank correlation coefficient (ρ). In addition, Pearson’s correlation coefficient (r) and the coefficient of determination (R^2^) were calculated to assess trend linearity and the proportion of explained variance. The strength of correlations was interpreted according to the Chaddock scale, which allows classification of the degree of association. Statistical significance was defined as *p* < 0.05. To quantitatively assess the association between sex and age groups, the odds ratio (OR) was calculated.

The correlation between seroprevalence estimates and incidence rates during 2014–2023 was evaluated using Spearman’s rank correlation coefficient. The analysis was based on data obtained from published studies as well as from the official federal statistical report “Information on Infectious and Parasitic Morbidity” [12,24,25,26]. The analysis included regions with stable diagnostic surveillance systems (Table 2). Data from the Udmurt Republic of Russia were excluded from the analysis because a statistical outlier (resulting from the combination of low Ab prevalence and high incidence) could have led to biased estimates and distortion of the final model [25]. Based on the remaining data, an average incidence-to-seroprevalence ratio across four regions was calculated. This averaged coefficient and the local seroprevalence values obtained for SPb and the LR allowed us to extrapolate incidence values.

Confidence intervals for proportions were calculated using the Wilson score interval with a 95% confidence level, which was preferred over the classical normal approximation (Wald interval) due to its greater robustness for moderate and low proportion values and for limited sample sizes in individual subgroups.

Statistical analyses were performed using the Python programming language (version 3.11.1) together with specialized libraries for statistical and mathematical computations, including SciPy (version 1.14.3), statsmodels (version 0.14.1), and pandas (version 2.2.3), which provide a comprehensive set of tools for data processing and analytical procedures.

Choropleth maps were generated using administrative boundary data. For the LR, boundary data were obtained from the GADM database (level 2). For SPb, an administrative boundary shapefile was manually constructed based on OpenStreetMap data. Data processing and visualization were conducted in the Python 3.10 environment using the GeoPandas (version 1.1.1) and Matplotlib (version 3.10.5) libraries. GADM data are distributed under the CC BY 4.0 license, and OpenStreetMap data are available under the ODbL 1.0 license.

## 3. Results

### 3.1. Seroprevalence of Hantaviruses in the Population of Saint Petersburg

**Figure 1:**
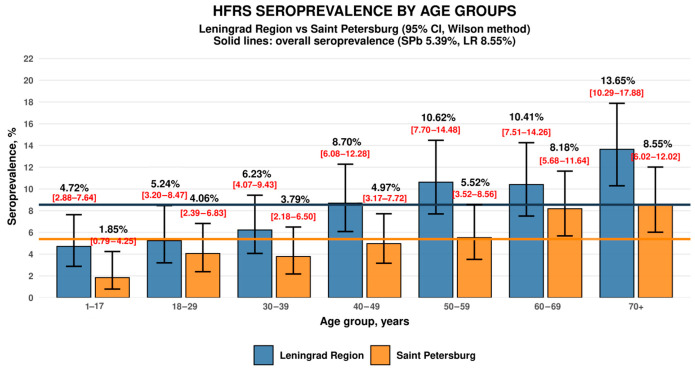
Hantavirus seroprevalence by age group within the overall study region. Vertical black lines are confidence intervals. Solid horizontal lines represent overall regional seroprevalence values: 5.39% (95% CI: 4.53–6.39) for St. Petersburg and 8.55% (95% CI: 7.45–9.75) for the LR.

For samples from SPb, hantavirus-specific IgG antibodies were detected in 122 out of 2265 examined volunteers, corresponding to a seroprevalence of 5.39% (95% CI: 4.53–6.39). An age-related increase in the proportion of seropositive individuals was observed, reaching statistical significance (χ^2^ = 18.89, *p* < 0.05, df = 6). The lowest seroprevalence was recorded in the 1–17-year age group, with a seroprevalence of 1.85% (95% CI: 0.79–4.25). No statistically significant differences were observed among the pediatric subgroups aged 1–5 years (0/51), 6–11 years (1/123), and 12–17 years (4/96) (*p* > 0.05, χ^2^ = 4.52). Therefore, subsequent comparative analyses were performed for the combined pediatric age group “1–17 years”. The highest proportion of seropositive individuals was observed in the age group ≥70 years, reaching 8.55% (95% CI: 6.02–12.02) (Figure 1). Statistical analysis revealed a significant, positive linear association between age and seroprevalence (hantavirus-specific IgG antibodies), as confirmed by Spearman’s rank correlation coefficient (ρ = 0.964), Pearson’s correlation coefficient (r = 0.924), and the coefficient of determination (R^2^ = 0.854). It is worth noting that the scientific literature reports a trend of increasing morbidity with age among women, a finding corroborated by the results of an epidemiological analysis conducted in SPb [27].

Following age-stratified analysis, sex-related differences in seroprevalence were assessed. Seroprevalence among men reached 7.59% (95% CI: 5.90–9.71), which was 1.8-fold higher than that observed among women, 4.29% (95% CI: 3.38–5.44). Non-overlapping confidence intervals confirmed the significance of these differences (χ^2^ = 10.84, *p* < 0.01, df = 1, OR = 1.83) (Table 3).

**Figure 2:**
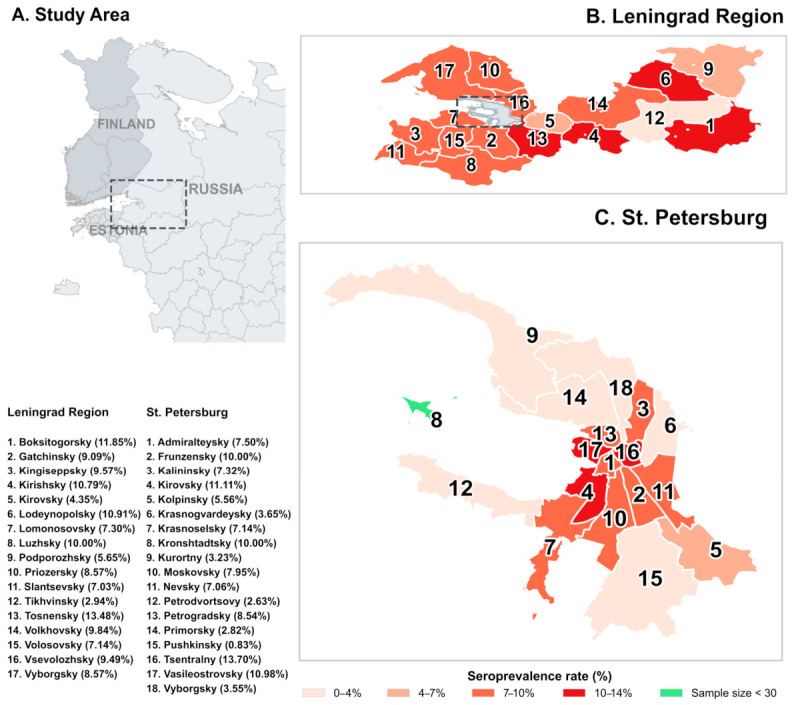
Hantavirus seroprevalence by district. Panel (**A**) shows study area. Panel (**B**) shows the Leningrad Region. Panel (**C**) shows Saint Petersburg.

Spatial analysis of seroprevalence across SPb districts (excluding the Kronstadt district) revealed the lowest level in the Pushkinsky district (0.83%, 95% CI: 0.45–2.25) and the highest in the Central district (13.70%, 95% CI: 7.61–23.41) (Table S1, Figure 2). However, no significant differences were detected among districts with the highest seroprevalence levels (χ^2^ = 0.76, *p* > 0.05, df = 3).

### 3.2. Seroprevalence of Hantaviruses in the Population of the Leningrad Region

The LR featured a higher seroprevalence than SPb. Hantavirus-specific IgG antibodies were detected in 188 out of 2199 individuals, corresponding to 8.55% (95% CI: 7.45–9.79). A similar age-related increase in seroprevalence was observed. The lowest value was recorded in the age group 1–17 years (4.72%, 95% CI: 2.88–7.64), while the highest was observed among individuals aged ≥70 years (13.65%, 95% CI: 10.29–17.88) (Figure 1). No significant differences were found among pediatric subgroups aged 1–5 years (3/62), 6–11 years (7/101), and 12–17 years (5/155) (*p* > 0.05, χ^2^ = 1.87). Therefore, the pediatric cohort in the LR was also analyzed as a combined age group (1–17 years). Analysis across all age groups revealed significant differences in seroprevalence (χ^2^ = 21.65, *p* < 0.01, df = 6). A strong, positive linear relationship between age and seroprevalence was identified, as confirmed by Spearman’s rank correlation coefficient (ρ = 0.964), Pearson’s correlation coefficient (r = 0.976), and the coefficient of determination (R^2^ = 0.953). Seroprevalence among children did not differ significantly from that observed in the age group of 18–29 years (5.24%, 95% CI: 3.20–8.47) (χ^2^ = 0.08, *p* = 0.77, df = 1, OR = 1.12). Across LR districts, seroprevalence ranged from 2.94% (95% CI: 1.96–8.72) in the Tikhvinsky district to 13.48% (95% CI: 8.80–20.09) in the Tosnensky district (Table S2, Figure 2).

Sex-related differences were also observed in the LR. Seroprevalence among men was 11.13% (95% CI: 8.83–13.94), whereas among women, it was 7.62% (95% CI: 6.42–9.01). These differences were significant (χ^2^ = 6.89, *p* < 0.05, df = 1, OR = 1.52) (Table 3).

## 4. Discussion

Our results reflect a discrepancy between the low level of officially registered HFRS incidence and the widespread presence of hantavirus-specific antibodies in the population of the examined regions. A comparison of the two regions revealed that the seroprevalence in the population of SPb is lower than that in the LR: 5.39% and 8.55%, respectively. The odds ratios for residents of the LR compared to SPb are OR = 1.64 (95% CI: 1.29–2.08, *p* < 0.001). This pattern is expected insofar as the population of the LR, unlike that of SPb, predominantly resides in private suburban houses. Furthermore, the LR has a considerable amount of forested areas; these provide favorable conditions for the natural habitation of rodents. In both SPb and the LR, a consistent age-related increase in seroprevalence was observed, suggesting cumulative exposure to hantaviruses over time. Statistically significant differences between age-related manifestations may be due to children having less contact with natural environments than adults. Adults, particularly those employed in agriculture, are at higher risk of infection due to their professional activities. These may involve regular contact with potential sources of infection, including wild mouse-like rodents, a reservoir of infection. The odds ratio comparing the oldest age group (≥70 years) with the pediatric group (1–17 years) was higher in SPb (OR = 4.94) than in the LR (OR = 3.18). The observed sex-related differences in seroprevalence in both regions may be attributable to differences in occupational and behavioral exposure [5,6]. Data from official registries indicate that hemorrhagic fever with renal syndrome (HFRS) predominantly affects working-age males, specifically those between 30 and 59 years old [28]. Men are more likely to be employed in settings with increased contact with rodents, such as food storage and livestock facilities. They also more frequently engage in outdoor and suburban activities, including fishing and hunting, which may increase the risk of hantavirus exposure.

Despite the relatively low mean annual incidence over the study period (≤1.0 per 100K population), the observed seroprevalence levels in both regions were substantial. These findings are consistent with previously reported serological data from HFRS-endemic Russian regions. For example, seroprevalence levels of 9.3 ± 0.4% were reported in the Tatarstan Republic of Russia during 2012–2021 [24], while values ranging from 7% to 20% were observed in the Bashkortostan Republic of Russia (2019–2020, 2022), and approximately 10% has been reported in the Udmurt Republic of Russia (2019) [25]. In Finland, which reports on of the highest HFRS incidence in Europe, seroprevalence has been shown to reach up to 12.5% according to recent data [26]. Assuming a direct relationship between disease incidence and seroprevalence, a significant correlation between these indicators was identified (Spearman’s ρ = 0.75, *p* = 0.05). The incidence-to-seroprevalence ratio (averaged across the four regions mentioned above) was calculated (K = 2.11). This coefficient and the local seroprevalence values obtained (SPb, LR) allowed us to extrapolate incidence values of 11.4 and 18.1 cases per 100K population, respectively. Given the limited number of regions included in this analysis (=4), these estimates should be considered preliminary and require confirmation in larger datasets.

The discrepancy between low officially registered incidence and relatively high seroprevalence may also reflect the circulation of mild or subclinical forms of HFRS. These may present without pronounced disease-specific symptoms, and they may be misclassified as other natural focal infections. Considering the evidence for long-lasting post-infectious immunity, it can be hypothesized that a substantial level of accumulated population immunity has developed in SPb and the LR over an extended period [29,30,31]. The presence of mild clinical forms of HFRS, which complicate diagnosis and registration of the disease, may be associated with genetic variants of hantaviruses circulating in the Northwestern Federal District, which requires further study of their genetic diversity [32]. The aforementioned highlights the need for further investigation of hantavirus genetic diversity.

## 5. Limitations

We acknowledge that additional methodological approaches are required to obtain more accurate results, and we recognize this as an important direction for future research. In the present study, we used a commercially available diagnostic kit. However, the manufacturer’s instructions did not specify the exact antigen used or the hantavirus types targeted by the kit. Furthermore, the distribution of volunteers across residential districts was uneven, as we were unable to recruit an equal number of participants from each district. Nevertheless, we included these data for illustrative and comparative purposes, as we believe that the geographic distribution of seroprevalence may reflect regional differences within SPb and LR and could prove useful for future epidemiological investigations.

## Figures and Tables

**Table 1:** Age structure of the volunteer cohort.

Age Group, Years		Number of Volunteers Examined		Total
		Saint Petersburg	Leningrad Region	
1–17		270	318	588 (13.17%)
subgroup	1–5	51	62	113 (19.21%)
	6–11	123	101	224 (38.10%)
	12–17	96	155	251 (42.69%)
18–29		320	286	606 (13.58%)
30–39		317	321	638 (14.29%)
40–49		362	322	684 (15.32%)
50–59		326	320	646 (14.47%)
60–69		330	317	647(14.49%)
≥70		340	315	655 (14.67%)
Total		2265	2199	4464 (100.00%)

**Table 2:** Summary of HFRS incidence and hantavirus seroprevalence data from recent studies in endemic regions. Data from publicly available sources over a multi-year period (2014–2023) are shown. The values listed were used for correlation analysis.

State/Region	Mean HFRS Incidence (Annual)	Seroprevalence, %
Finland	23.85	12.50
Kostromsky Region	14.77	10.00
Bashkortostan Republic	35.26	13.00
Tatarstan Republic	18.62	9.30
Udmurt Republic	56.85	10.00

**Table 3:** Gender distribution of hantavirus seropositivity.

Region	Results	Male	Female
Saint Petersburg	IgG positive/total tested	57/751	65/1514
	Seroprevalence, % (95% CI)	7.59 (5.90–9.71)	4.29 (3.38–5.44)
Leningrad Region	IgG positive/total tested	65/584	123/1615
	Seroprevalence, % (95% CI)	11.13 (8.83–13.94)	7.62 (6.42–9.01)
Total	IgG positive/total tested	122/1335	188/3129
	Seroprevalence, % (95% CI)	9.14 (7.71–10.80)	6.01 (5.23–6.90)

## Data Availability

The original contributions presented in this study are included in the article/Supplementary Materials. Further inquiries can be directed to the corresponding author.

## Supplementary Materials

The following supporting information can be downloaded at: https://www.mdpi.com/article/10.3390/v18060652/s1, Table S1: Hantavirus seroprevalence by Saint Petersburg district; Table S2: Hantavirus seroprevalence by Leningrad Region district.
