# Supplementary material for: Hantavirus Seroprevalence in the Population of Saint Petersburg and the Leningrad Region, Russia
Source: Viruses. 2026 Jun 6;18(6):652. doi: 10.3390/v18060652 (PMC13307824; doi:10.3390/v18060652)
Supplement: Supplementary file 1 [file viruses-18-00652-s001.zip › viruses-4345626-supplementary.pdf]

Table S1. Hantavirus seroprevalence by Saint Petersburg district.

District	IgG positive/total tested	Seroprevalence, %	(95% CI)
Admiralteysky	6/80	7.50	(3.48–15.41)
Vasileostrovsky	9/82	10.98	(5.88–19.56)
Vyborgsky	15/422	3.55	(2.16–5.78)
Kalininsky	6/82	7.32	(3.39–15.05)
Kirovsky	9/81	11.11	(5.96–19.79)
Kolpinsky	3/54	5.56	(1.90–15.11)
Krasnogvardeisky	8/219	3.65	(1.86–7.04)
Krasnoselsky	10/140	7.14	(3.92–12.65)
Kronstadtsky	1/10	10.00	(1.79–40.42)
Kurortny	1/31	3.23	(0.57–16.19)
Moskovsky	7/88	7.95	(3.90–15.52)
Nevsky	6/85	7.06	(3.27–14.56)
Petrogradsky	7/82	8.54	(4.19–16.59)
Petrodvortsovy	1/38	2.63	(0.46–13.49)
Primorsky	14/497	2.82	(1.68–4.67)
Pushkinsky	1/121	0.83	(0.45–2.25)
Frunzensky	8/80	10.00	(5.15–18.51)
Tsentralny	10/73	13.70	(7.61–23.41)
**Total**	**122/2265**	**5.39**	**(4.53–6.39)**

Table S2. Hantavirus seroprevalence by Leningrad Region district.

District	IgG positive/total tested	Seroprevalence, %	(95% CI)
Boksitogorsky	16/135	11.85	(8.05–17.93)
Volosovsky	10/140	7.14	(4.29–12.82)
Volkhovsky	12/122	9.84	(6.52–17.95)
Vsevolozhsky	13/137	9.49	(6.28–17.28)
Vyborgsky	12/140	8.57	(5.82–16.07)
Gatchinsky	12/132	9.09	(6.06–16.74)
Kingiseppsky	11/115	9.57	(5.44–16.77)
Kirishsky	15/139	10.79	(7.69–17.36)
Kirovsky	6/138	4.35	(2.94–10.97)
Lomonosovsky	10/137	7.30	(5.14–15.81)
Lodeynopolsky	6/55	10.91	(5.01–20.94)
Luzhsky	14/140	10.00	(6.28–17.28)
Priozersky	12/140	8.57	(5.35–15.98)
Podporozhsky	7/124	5.65	(3.94–12.97)
Slantsevsky	9/128	7.03	(4.88–14.88)
Tikhvinsky	4/136	2.94	(1.96–8.72)
Tosnensky	19/141	13.48	(8.84–18.58)
**Total**	**188/2199**	**8.55**	**(7.45–9.75)**
